# Lobaplatin-based prophylactic hyperthermic intraperitoneal chemotherapy for T4 gastric cancer patients: A retrospective clinical study

**DOI:** 10.3389/fonc.2023.995618

**Published:** 2023-01-18

**Authors:** Yuxin Zhong, Wenzhe Kang, Haitao Hu, Weikun Li, Jing Zhang, Yantao Tian

**Affiliations:** ^1^ Department of Pancreatic and Gastric Surgery, National Cancer Center/National Clinical Research Center for Cancer/Cancer Hospital, Chinese Academy of Medical Sciences and Peking Union Medical College, Beijing, China; ^2^ Department of Surgery, Huanxing Cancer Hospital, Beijing, China

**Keywords:** gastric cancer, lobaplatin, hepatic intraperitoneal chemotherapy, prognosis, advanced

## Abstract

**Objective:**

To explore the clinical efficacy of lobaplatin-based prophylactic hyperthermic intraperitoneal chemotherapy (HIPEC) for patients with T4 gastric cancer after surgery and to evaluate its impact on survival.

**Materials and methods:**

Data on patients with T4 gastric cancer who underwent radical gastric resection between March 2016 and August 2017 were collected from the National Cancer Center and Huangxing Cancer Hospital. Enrolled patients were divided into two groups according to receiving or not receiving HIPEC.

**Results:**

A total of 106 patients were included in this study; among them, 51 patients underwent radical gastric resection plus prophylactic HIPEC, and 55 patients underwent radical gastric resection only. The baseline characteristics were well balanced between the two groups. The postoperative platelet counts in the HIPEC group were significantly lower than those in the non-HIPEC group (P < 0.05); however, we did not observe any occurrences of serious bleeding in the HIPEC group. There were no significant differences in the postoperative complication rates between the two groups (P > 0.05). The postoperative (1 month) CEA, CA19-9, and CA72-4 levels in the HIPEC group were significantly decreased in the HIPEC group (P < 0.05). At a median follow-up of 59.3 months, 3 (5.5%) patients in the HIPEC group experienced peritoneal recurrence, and 10 (18.2%) patients in the non-HIPEC group experienced peritoneal recurrence (P < 0.05). Both groups had comparable 5-year overall survival (OS) rates (41.1% HIPEC group *vs*. 34.5% non-HIPEC group, P = 0.118). The 5-year disease-free survival was significantly higher in the HIPEC group than in the non-HIPEC group (28.6% versus 39.7%, p = 0.046).

**Conclusions:**

Lobaplatin-based prophylactic HIPEC is feasible and safe for patients with T4 gastric cancer and does not increase postoperative adverse effects. The use of HIPEC showed a significant decrease in the incidence of local recurrence rates and blood tumor marker levels. The 5-year disease-free survival was significantly higher in the HIPEC group; however, the 5-year OS benefit was not found in T4 stage patients.

## 1 Introduction

According to the 2020 Global Cancer Statistics Report, gastric cancer (GC) has become the fifth most common cancer worldwide and has the fourth highest mortality rate. It is estimated that there were over one million new gastric cancer cases and approximately 770,000 gastric cancer deaths in 2020 (1). In China, nearly 78% of the cases are diagnosed as advanced gastric cancer at the time of the initial clinical visit (2). Surgical resection combined with chemotherapy is the primary treatment for advanced gastric cancer, but the therapeutic effect is not satisfactory (3). Peritoneal metastasis is one of the most common and important causes of poor prognosis for gastric cancer patients with serosal involvement (T4), and it accounts for nearly half of the postoperative relapses (4).

Metastasis to the peritoneum can lead to several complications, such as ascites, bowel obstruction, and hydronephrosis, all of which worsen the patient’s condition and quality of life (5–7).

In recent years, HIPEC has been used as one form of treatment for patients with advanced gastrointestinal cancers (8, 9). Compared with conventional intravenous chemotherapy, HIPEC has significant advantages (10). It increases the amount of chemotherapeutics within the tumor cells and, coupled with thermal effects, can kill tumor cells (11). Lobaplatin, as a third generation platinum-based anticancer agent, exerts stronger antitumor activity, with lower toxicity and higher solubility and stability in water (12). Lobaplatin-based HIPEC has been gradually applied to treat advanced gastric cancer after radical surgery to reduce peritoneal metastasis (13). To determine the safety and efficacy of radical surgery with prophylactic lobaplatin-based HIPEC, especially its effect on peritoneum metastasis and long-term survival, we retrospectively analyzed the clinical data of patients with T4 gastric cancer admitted to the National Cancer Center and Huangxing Cancer Hospital.

## 2 Materials and methods

### 2.1 Study population and grouping

We retrospectively reviewed the clinical data of patients with gastric cancer who underwent radical surgical resection at the National Cancer Center and Huangxing Cancer Hospital between March 2016 and August 2017. The surgeon explained the possible benefits and risks to the cT4 gastric cancer patients to obtain their informed consent, and the patients decided whether or not receiving HIPEC by themselves. Before surgery, hematological examination, contrast-enhanced computed tomography (CT), gastroscopy were routinely performed on all patients for tumor staging. And If lymph node metastasis was suspected based on preoperative imaging, neoadjuvant chemotherapy was performed. The patients were divided into two groups according to whether prophylactic HIPEC was performed. This study was conducted following the ethical standards of the Declaration of Helsinki and within the national and international guidelines. The study was performed with ethics approval from the Ethics Committee of National Cancer Center, Chinese Academy of Medical Sciences (NCC2017-YZ-026). Due to the retrospective nature of this study, consent of the patients to review their medical records was not required by the Institutional Review Board (IRB). The inclusion criteria were as follows: 1. Pathologically diagnosed as gastric adenocarcinoma; 2. The postoperative pathological stage was T4, with or without lymph node metastasis; 3. Absence of distant metastasis (M0); 4. Age between 18 and 75 years old; 5. The laboratory examinations had to meet the following conditions: white blood cells ≥ 4.0×109/L; neutrophil counts ≥ 2.0×109/L; platelets ≥ 100×109/L; hemoglobin≥90 g/L; serum total bilirubin ≤ 17.1 μmol/L; serum alanine aminotransferase ≤ 1.5×ULN; serum creatinine ≤ 1.5×ULN; creatinine clearance ≥ 60 ml/min (Cockcroft-Gault formula). The exclusion criteria included the following: 1. Tumor located at esophagogastric junction, and thoracotomy was needed; 2. Distant metastasis (M1), including peritoneal metastasis; 3. Patients with severe complications who could not tolerate or who refused prophylactic HIPEC; 4. Patients with previous or coexisting other malignant diseases; 5. Patients who had a history of neurological or psychiatric disease and who were unable to complete the follow-up evaluation. Finally, 106 patients with gastric cancer met the inclusion criteria. Among them, 51 patients were included in the radical surgery plus prophylactic HIPEC group, and 56 patients underwent radical surgery.

### 2.2 Treatment method

The patients in the two groups underwent laparoscopic-assisted radical gastrectomy. The patients were placed under general anesthesia with orotracheal intubation. The patients were placed in a supine position with their legs split, the surgeon operated from the right side, the assistant was on the left side and the camera assistant was between the patients’ legs. Laparoscopic radical surgery was performed with a five-hole approach. Based on the tumor location, total or distal subtotal gastrectomy with D2 lymphadenectomy was performed. After transection, a 5-7 cm long incision was made in the mid-upper abdomen, the specimen was removed, and extracorporeal digestive tract reconstruction was performed through the incision. Reconstruction of the digestive tract included Billroth-I, Billroth-II, and Roux-en-Y after gastrectomy.

In the HIPEC group, lobaplatin-based prophylactic HIPEC was performed under general anesthesia after closure of the incision. Two inlet pipes and two outlet pipes were installed. Lobaplatin (50mg/m^2^) was diluted in heated 5% glucose solution and then was circulated for 60 min. The perfusion rate was 400-500 ml/min. The circulating temperature was maintained at 42.5-43°C. After HIPEC, at least 90% of the perfusion fluid was removed. The patient’s vital signs and color of drainage were observed carefully during HIPEC. All patients received adjuvant chemotherapy after radical surgical resection.

### 2.3 Postoperative follow-up

The first day after surgery represented the beginning of the follow-up period. According to the NCCN gastric cancer guidelines, the patients were followed every 3 months for the first two years, then every 6 months for the next three years and then once a year after five years. The follow-up examinations included biochemical tests, tumor marker examination, and computed tomography (CT). The last date of follow-up was April 7^th^, 2022. Disease-free survival (DFS) was defined as the time from surgery to recurrence, while overall survival (OS) was defined as the time from surgery to all deaths.

### 2.4 Statistical analysis

Data statistics and analysis were conducted using Statistical Package for the Social Sciences (SPSS) version 23.0 for Windows (IBM Corp, Armonk, NY, United States) and GraphPad Prism (version 8, GraphPad Prism Software Inc.). Continuous data are expressed as the mean ± SD and were analyzed by a t test. Categorical data are shown as frequencies and percentages and were analyzed by the chi-squared test or Fisher’s exact test. Ranked and abnormally distributed quantitative data were assessed by the Mann−Whitney test. Survival analysis was performed using Kaplan−Meier curves and the log-rank test. Differences were considered significant when the P value was less than 0.05.

## 3 Results

### 3.1 Patient characteristics

A total of 106 patients met our inclusion criteria, and they had a mean age of 54.2 ± 10.3 years. Of these patients, 51 (48.1%) patients were in the HIPEC group, and 55 (52.9%) patients were in the non-HIPEC group. These two groups of patients were well balanced in terms of age, sex, ASA score, BMI, gastrectomy, tumor grade and pathological N staging (**Table 1**). The mean operation time in the HIPEC group was significantly longer than that in the non-HIPEC group (206 ± 35.6 min *vs*. 164 ± 34.3 min, p = 0.034). There were no significant differences in conversion to open surgery, estimated blood loss or hospital stay after the operations. Similarly, the postoperative gastrointestinal recovery was not significantly different between the two groups regarding the time to first flatus (2.3 ± 1.6 days versus 2.2 ± 1.2 days, p = 0.744) and time to regular diet (5.4 ± 2.2 days versus 5.2 ± 2.9 days, p = 0.649). Five (9.8%) patients in the HIPEC group had abnormal liver function (elevated ALT level), and 2 (3.6%) patients in the non-HIPEC group had abnormal liver function. All patients recovered to have a normal liver function after being given liver protection drugs. Nine (17.6%) patients in the HIPEC group suffered a decline in their peripheral platelet count (less than 150,000 platelets per microliter), which included 2 (3.6%) patients in the non-HIPEC group (p = 0.026); however, none of the patients in either of the groups experienced major bleeding events leading to anemia. There were no cases of 30-day postoperative mortality in either group (**Table 2**).

**Table 1:** Baseline characteristics of the T4 gastric cancer patients.

Characteristics	All patients	HIPEC	Non-HIPEC	*P value*
	(n = 106)	(n = 51)	(n = 55)	
Age at diagnosis, year, (n%)	54.2 ± 10.3	53.5 ± 10.5	55.1 ± 10.9	0.252
21-49	22 (20.8%)	12 (23.5%)	10 (18.2%)	
50-75	71 (67.0%)	34 (66.7%)	37 (67.3%)	
76-96	13 (10.2%)	5 (9.8%)	8 (14.5%)	
Sex, n (%)				0.748
Female	49 (46.2%)	24 (47.1%)	25 (45.5%)	
Male	57 (53.8%)	27 (52.9%)	30 (54.5%)	
ASA score				0.275
I	34 (32.1%)	16 (31.4%)	18 (32.7%)	
II	68 (64.2%)	33 (64.7%)	35 (63.6%)	
III	4 (3.7%)	2 (3.9%)	2 (3.6%)	
BMI, kg/m2 (mean ± SD)	24.2 ± 2.5	23.6 ± 2.4	25.5 ± 2.7	0.334
Gastrectomy				0.438
Proximal	18 (17.0%)	10 (19.7%)	8 (14.5%)	
Distal	72 (67.9%)	33 (64.7%)	39 (71.0%)	
Total	16 (15.1%)	8 (15.6%)	8 (14.5%)	
Tumor grade				0.458
Poor or moderately	67 (63.2%)	33 (64.7%)	34 (61.8%)	
Mucinous or signet cell	39 (36.8%)	18 (35.3%)	21 (38.2%)	
Neoadjuvant CHT				0.347
Yes	82 (77.4%)	39 (76.5%)	43 (78.2%)	
No	24 (22.6%)	12 (23.5%)	12 (21.8%)	
Pathological N staging				0.354
N0	10 (9.4%)	5 (9.8%)	6 (10.9%)	
N1	39 (36.8%)	18 (35.3%)	21 (38.2%)	
N2	46 (43.4%)	24 (47.1%)	22 (40%)	
N3	11 (10.4%)	5 (9.8%)	6 (10.9%)	

**Table 2:** Perioperative data of the patients.

Characteristics	HIPEC	Non-HIPEC	*P value*
	(n = 51)	(n = 55)	
Conversion to open, n (%)	2 (3.9%)	3 (5.5%)	1
Operation time in min, mean ± SD	206 ± 35.6	164 ± 34.3	0.034
Estimated blood loss in mL, mean ± SD	85.2 ± 23.6	77.9 ± 25.8	0.553
Hospital stay after operation (d, mean ± SD)	7.1 ± 1.3	7.7 ± 0.9	0.386
30 d post-operative mortality, n (%)	0	0	N/A
Time to first flatus, day (mean±SD)	2.3 ± 1.6	2.2 ± 1.2	0.744
Time to Regular diet, day (mean±SD)	5.4 ± 2.2	5.2 ± 2.9	0.649
Postoperative complications (grades III, IV)			
Anastomotic Leakage	2 (3.9%)	1 (1.8%)	0.704
Bowel Obstruction	2 (3.9%)	2 (3.6%)	1
Surgical Wound Infection	5 (9.8%)	6 (10.9%)	0.833
Bleeding	2 (3.9%)	3 (5.5%)	0.936
Delayed gastric emptying	5 (9.8%)	7 (12.7%)	0.557
Lung infection	1 (2.0%)	0	1
Fever	6 (11.8%)	3 (5.5%)	0.129
Abnormal blood routine tests	9 (17.6%)	2 (3.6%)	0.026
Abnormal renal function	1 (2.0%)	1 (1.8%)	0.957
Abnormal liver function	5 (9.8%)	2 (3.6%)	0.068
Severe neurotoxicity	0	0	N/A
Peritoneal recurrence	3 (5.5%)	10 (18.2%)	0.037

The CEA positive rate, CA199 positive rate and CA724 positive rate 1 month after surgery in the HIPEC group were significantly lower than those in the non-HIPEC group (23.53% versus 47.27%, p = 0.044; 23.50% versus 43.64%, p = 0.049; 31.37% versus 52.73%, p = 0.025). There were no significant differences in terms of the CA125 positive rate between the two groups (21.57% versus 32.73%, p = 0.063) (**Table 3**).

**Table 3:** Comparison of the tumor markers positive rates between the two groups before surgery and 1 month after surgery.

Characteristics	HIPEC	Non-HIPEC	*P value*
	(n = 51)	(n = 55)	
**CEA positive rate, (n%)**			0.044
Before surgery	90.20%	89.10%	
1month after surgery	23.53%	47.27%	
**CA199 positive rate, (n%)**			0.049
Before surgery	88.00%	85.45%	
1month after surgery	25.50%	43.64%	
**CA724 positive rate, (n%)**			0.025
Before surgery	86.37%	87.27%	
1month after surgery	31.37%	52.73%	
**CA125 positive rate, (n%)**			0.063
Before surgery	84.31%	81.82%	
1 month after surgery	21.57%	32.73%	

### 3.2 Survival analysis

**Figure 1:**
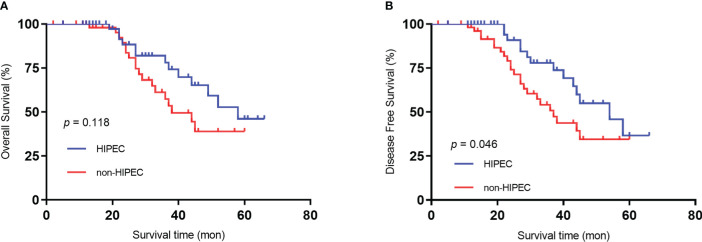
Kaplan‒Meier survival curves depicting the survival in patients with T4 gastric cancer. **(A)** Patient OS based on whether HIPEC was performed. **(B)** Patient DFS based on whether HIPEC was performed..

The mean follow-up time was 59.3 months. All patients underwent postoperative adjuvant chemotherapy (oxaliplatin plus capecitabine for 6 cycles). During the follow-up period, 3 (5.5%) patients in the HIPEC group experienced peritoneal recurrence, and 10 (18.2%) patients in the non-HIPEC group experienced recurrence (p < 0.05) (**Table 2**). Both groups had comparable 5-year overall survival (OS) rates (41.1% HIPEC group *vs*. 34.5% non-HIPEC group, P = 0.118) (**Figure 1A**). The 5-year disease-free survival was significantly higher in the HIPEC group than in the non-HIPEC group (39.7% versus 28.6%, p = 0.046) (**Figure 1B**). A multivariable analysis was performed using logistic regression to determine factors associated with disease-free survival. The results showed that poorly differentiated, mucinous or signet cell adenocarcinoma, pathological positive lymph nodes and no neoadjuvant chemotherapy were significantly associated with poor disease-free survival. On the other hand, patients who underwent radical surgery plus HIPEC were less likely to have tumor recurrence (**Table 4**).

**Table 4:** Multivariable analysis of the prognostic factors influencing DFS.

Characteristics	OR (95% CI)	*P* value
Age at diagnosis, year		
21-49	1 [Reference]	
50-75	0.933 (0.723-1.235)	0.527
76-96	0.622 (0.378-1.254)	0.054
Sex		
Female	1 [Reference]	
Male	1.216 (0.873-1.345)	0.343
ASA score		
I	1 [Reference]	
II	0.733 (0.485-1.176)	0.14
III	1.340 (0.842-1.875)	0.31
Gastrectomy		
Proximal	1 [Reference]	
Distal	0.777 (0.581-1.040)	0.09
Total	0.850 (0.642-1.125)	0.255
Tumor grade		
Poor or moderately	1 [Reference]	
Mucinous or signet cell	1.521 (1.113-2.124)	0.005
Pathological N staging		
N0	1 [Reference]	
N1/N2/N3	1.347 (1.054-1.674)	0.038
Neoadjuvant chemotherapy		
Yes	1 [Reference]	
No	1.467 (1.026-1.772)	0.024
Treatment modality		
Surgery	1 [Reference]	
Surgery plus prophylactic HIPEC	0.584 (0.348-0.837)	0.046
Preoperative abnormal tumor markers level		
CEA levels	1.207 (0.984-1.326)	0.674
CA199 levels	1.237 (1.014-1.528)	0.437
CA125 levels	1.207 (0.835-1.429)	0.348
CA724 levels	1.372 (0.934-1.539)	0.332

## 4 Discussion

In this double-center retrospective observational study, we found that in patients with T4 gastric cancer, prophylactic HIPEC was a strong independent positive predictor of DFS. Patients who underwent prophylactic HIPEC had a lower peritoneal recurrence rate. The results also indicated that the expression of tumor markers 1 month after surgery was decreased significantly in the HIPEC group. However, these advantages did not translate into an OS benefit.

Whether prophylactic PIPEC in patients with T4 gastric cancer affords a survival advantage is still controversial, and research in this area has remained rather limited. There is still a lack of a unified treatment strategy, and no consensus is available regarding this topic. Radical surgery and HIPEC were first described in 1980 (14), and some of the previous studies and meta-analyses reported that the incidence of postoperative complications was higher in the HIPEC group (15–17) (18–21). In 2000, Samel et al. (18) reported 9 patients with advanced gastric cancer who underwent gastrectomy plus intraoperative HIPEC. The results showed that 6 of the patients (66%) developed postoperative complications, including anastomotic leakage, pancreatic fistula, pancreatitis and renal failure, indicating that intraoperative HIPEC was associated with a high risk of perioperative complications. French researchers (19) conducted a retrospective multicenter study of 159 patients from 15 institutions. A total of 10 (6.5%) patients died after surgery. The causes of death included multiple organ failure (2 patients), septic shock (2 patients), respiratory complications (2 patients), peritonitis caused by anastomotic leakage (1 patient), thromboembolic events (1 patient), cardiac arrhythmia (1 patient), and hematological toxicity (1 patient). The postoperative grade 3-4 morbidity rate was 27.8%. Fourteen percent of the patients required surgery. They concluded that for patients with gastric cancer, surgery with HIPEC may achieve long-term survival in a selected group of patients. Because of its high mortality rate, a stringent screening process should be employed and should be reserved for experienced institutions. The high overall complication rate in the past was probably due to the immature method of implementation of HIPEC, technological imperfections, and inaccurate temperature and may also be related to the general physical conditions of patients (20–22).

With the continuous exploration of HIPEC and improvement of its instruments, the application of HIPEC in gastrointestinal tumors has been quite mature (23). In recent years, a number of studies have suggested that HIPEC does not increase the incidence of postoperative complications (24–28). Beeharry et al. (29) conducted a randomized case−control study in 2019, and 80 patients with locally advanced gastric cancer were randomly divided into two groups: the HIPEC group (curative resection plus intraoperative HIPEC) and the control group (curative resection only). Their results indicated that faster recovery of bowel function (43 ± 5 h *vs*. 68 ± 7, P < 0.05) and shorter postoperative stay (8 d *vs*. 14 d, P < 0.05) were noted in the HIPEC group. Mild renal dysfunction, mild liver dysfunction and hyperbilirubinemia were recorded in the HIPEC group, but their incidences were found to be statistically insignificant when compared with the control group (P > 0.05). The prophylactic HIPEC group had a higher DFS rate and a lower peritoneal recurrence rate. These conclusions are similar to our conclusions.

Our conclusion demonstrated that thrombocytopenia occurred in 9 (17.6%) patients in the HIPEC group, which was significantly higher than that in the non-HIPEC group (n = 2, 3.6%). However, no severe bleeding events occurred in either group. Even then, liver and renal function, as well as routine blood tests, must be closely monitored in patients who undergo HIPEC after surgery. Our results also showed that there were no significant differences between the two groups in terms of complications and adverse events; this result is consistent with recent research.

Our results also indicated that after one month of treatment, the positive rates of tumor markers (CEA, CA199, CA724) were significantly lower in the HIPEC plus surgery group than in the CRS alone group. Both treatment strategies are helpful to reduce the level of serum tumor markers, while HIPEC plus surgery is more effective.

In this study, the results showed that patients with T4 gastric cancer who had HIPEC after surgery had better DFS and a lower peritoneal recurrence rate but showed no significant benefit in OS. The multivariable analysis confirmed that HIPEC had a significant and positive impact on DFS. At present, some studies are consistent with this result. Desiderio et al. (30) published a meta-analysis in 2017, and a total of 32 trials (2520 patients) were included. The analysis showed that HIPEC had advantages in preventing peritoneal metastasis, and the 3-year OS rate and 5-year OS rate in the HIPEC group were significantly higher than those in the non-HIPEC group (29).

This study has several limitations. First, even though the clinicopathological characteristics of the two groups were balanced, the retrospective nature of this study may have led to selection bias, and there might be unknown confounders that could have affected the results of this study. Second, this was a double-center study, and the sample size was relatively small. Multicenter, large-scale, randomized studies are needed to further confirm the survival benefits provided by lobaplatin-based prophylactic HIPEC in patients with T4 gastric cancer.

In conclusion, our study shows that lobaplatin-based prophylactic HIPEC is feasible and safe for patients with T4 gastric cancer and does not increase postoperative adverse effects. The use of HIPEC was associated with a significantly decreased incidence of peritoneal recurrence rates and blood tumor marker levels. The 5-year disease-free survival was significantly higher in the HIPEC group; however, a 5-year OS benefit was not found in T4 stage patients.

## Data availability statement

The raw data supporting the conclusions of this article will be made available by the authors, without undue reservation.

## Author contributions

YZ and WK: acquisition of data. YZ: analysis and interpretation of data. YZ, HH and WL: drafted the manuscript. JZ and YT: critical revision of the manuscript. All authors contributed to the article and approved the submitted version.
